# Dysglycaemia is associated with the pattern of valvular calcification in micro-computed tomography analysis: an observational study in patients with severe aortic stenosis

**DOI:** 10.1186/s12933-025-02691-y

**Published:** 2025-03-20

**Authors:** Magdalena Kopytek, Kamila W. Undas, Jacek Tarasiuk, Sebastian Wroński, Michał Ząbczyk, Joanna Natorska

**Affiliations:** 1 Department of Thromboembolic Disorders, Institute of Cardiology, Jagiellonian University Medical College, Krakow, Poland; 2 Krakow Centre for Medical Research and Technologies, St. John Paul II Hospital, Krakow, Poland; 3 Faculty of Medicine, Jagiellonian University Medical College, Krakow, Poland; 4 Faculty of Physics, Astronomy and Applied Computer Science, Jagiellonian University, Krakow, Poland; 5 Department of Condensed Matter Physics, Faculty of Physics and Applied Computer Science, AGH University of Krakow, Krakow, Poland

**Keywords:** Aortic stenosis, Pre-diabetes, Impaired glucose, Glycated haemoglobin, Advanced glycation end products, Micro-computed tomography, Calcification, Mineralization

## Abstract

**Background:**

Diabetes mellitus (DM) has been shown to increase the rate of aortic stenosis (AS) progression. However, the impact of impaired plasma glucose on valvular calcification remains poorly understood. Using ex vivo micro-computed tomography (micro-CT), we aimed to determine whether plasma glucose, glycated haemoglobin (HbA_1c_), or concentrations of advanced glycation end products (AGEs) and their soluble receptor (sRAGE) are associated with a specific pattern of valvular calcification in severe AS.

**Methods:**

In this case-control study, 14 (48%) normoglycaemic patients with AS were compared to 15 individuals (52%) with elevated glucose levels (≥ 5.6 mmol/L), all with HbA_1c_ ≤ 6.5%. Stenotic aortic valves obtained surgically were analysed using micro-CT to assess structure of tissue mineralization. Calcium volume (CV), surface volume (SV), CV/SV ratio, and trabecular thickness (TbTh) were evaluated. Plasma AGEs and sRAGE were assessed by ELISAs. DM patients or those using antidiabetic agents were excluded from the study.

**Results:**

Patients with impaired and high glucose, including 10 (67%) with glucose between 5.6 and 6.9 mmol/L and 5 (33%) ranging from 7 to 7.6 mmol/L, exhibited higher HbA_1c_ (+ 17%) and AGEs levels (+ 44.6%), but not sRAGE compared to those with normal glucose. Patients with impaired and high glucose had also 19.2% higher maximal transvalvular pressure gradient (PG_max_) and 9.3% higher peak transvalvular velocity (V_max_) compared to normoglycaemic individuals. Micro-CT indices correlated with fasting glucose, HbA_1c_, and AGEs levels (all *p* < 0.05), but not with sRAGE (*p* > 0.05). Valves extracted from patients with impaired and high glucose exhibited higher mineralization volume, folding, and structural integrity, as reflected by increased CV (+ 127.6%), CV/SV ratio (+ 59%) and calcium deposits microarchitecture as indicated by about 50% higher TbTh, compared to normoglycaemic patients. When patients with AS were divided into three groups based on their glucose levels (< 5.5 mmol/L, 5.6–6.9 mmol/L, and 7.0–7.6 mmol/L), micro-CT analysis showed more distinct structural differences among the groups. The valves in the highest glucose group were the most severely affected. Micro-CT parameters were also associated with both transvalvular pressure gradients (PG_mean_ and PG_max_), V_max_ and aortic valve area (all *p* < 0.05).

**Conclusions:**

Strict glycaemic control could potentially reduce the rate of valve mineralization and calcium deposit accumulation in patients with AS.

**Graphical abstract:**

Pre-diabetic AS patients showed greater AS severity as measured by echocardiography, increased AGEs concentrations, and increased valvular calcification. Micro-CT parameters correlated with fasting glucose, HbA_1c_, AGEs concentrations, and disease severity.

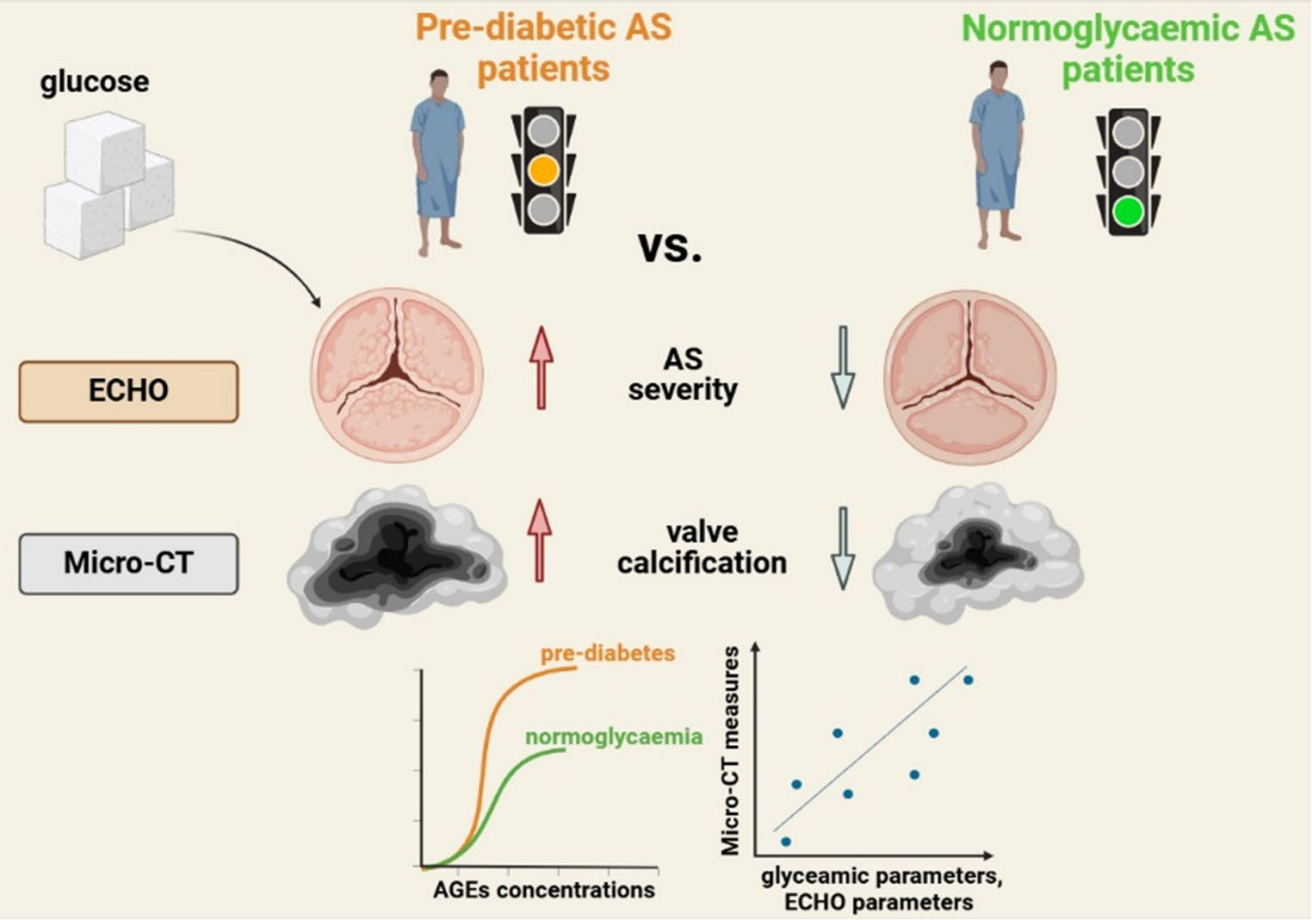

**Research insights:**

**What is currently known about this topic?:**

Diabetes mellitus (DM) is a risk factor for the progression of aortic stenosis (AS). Accumulation of advanced glycation end products (AGEs) enhances glycation of valvular proteins.

**What is the key research question?:**

Is dysglycaemia associated with more severe aortic valve calcification in patients with severe AS? Is ex vivo micro-CT suitable for assessing differences in calcification pattern within stenoticvalves?

**What is new?:**

Pre-diabetic patients with AS show increased valvular calcium volume, surface corrugation, and calcium deposit integrity. Micro-CT parameters associate with glycaemic status and echocardiographic measures of AS severity. Micro-CT provides precise assessment of calcification, offering insights beyond traditional methods.

**How might this study influence clinical practice?:**

Strict glycaemic control together with CT calcium scoring should be performed in patients with AS to monitor disease progression.

**Supplementary Information:**

The online version contains supplementary material available at 10.1186/s12933-025-02691-y.

## Introduction

Aortic stenosis (AS), the third most prevalent cardiovascular condition in developed countries [1], is a disease of considerable epidemiological importance. Its incidence rises with age, exceeding 1,000 cases per 100,000 individuals over 75 years old, and currently affects 9.4 million patients [2]. To date, no pharmacological treatment has been developed to reverse or slow down the rate of AS progression. The only therapeutic options currently available for managing AS are surgical aortic valve replacement (SAVR) or transcatheter aortic valve replacement (TAVR). AS is a condition characterized by the thickening of the aortic leaflets and obstruction of left ventricular outflow mainly due to calcification, leading to adverse hemodynamic changes [3].

Of note, AS shares several pathophysiological mechanisms with atherosclerosis [3]. Diabetes mellitus (DM) is a well-established risk factor for both atherosclerotic vascular disease and AS [4, 5]. However, the effect of hyperglycaemia on valvular inflammation and calcification remains poorly understood [5–9]. It has been demonstrated that elevated expression of nuclear factor-κB (NF-κB) in diabetic AS patients is associated with increased valvular expression of the calcification marker bone morphogenetic protein 2 (BMP-2), especially in subjects with poorly controlled DM, defined as glycated haemoglobin (HbA_1c_) ≥ 6.5% [9]. In vitro studies supported these findings and demonstrated that high doses of glucose activated oxidative stress and pro-inflammatory state in valvular interstitial cells (VICs), while inhibition of reactive oxygen species or NF-κB pathways was shown to prevent valvular calcification [9]. Additionally, the accumulation of advanced glycation end products (AGEs), leading to increased glycation of valvular proteins, has been proposed as a factor contributing to the accelerated AS progression [8, 10, 11]. Diabetic AS patients exhibited elevated levels of both valvular and plasma AGEs, which correlated with disease severity as measured by echocardiography [8]. Of note, in patients with well-controlled DM, the effect of hyperglycemia on AS severity appeared minimal [8]. Interestingly, a recent study by Hu et al. [12] demonstrated that the stress hyperglycemia ratio, which considers both admission plasma glucose and HbA_1c_ levels, was associated with all-cause mortality, cardiovascular mortality, readmission due to heart failure, and major adverse cardiovascular events (MACE) in patients with severe AS undergoing TAVR. Therefore, research exploring the impact of short- and long-term glycemic control variables on valvular calcification is warranted.

Computed tomography (CT) due to relatively low resolution remains insufficient for an in-depth calcium deposit pattern evaluation in vivo. We have developed the ex vivo micro-CT imaging [13], which yields high-quality images enabling more precise calcification pattern examination.

The aim of our study was to evaluate using ex vivo micro-CT analysis whether impaired plasma glucose levels, HbA_1c_, or plasma concentrations of AGEs are associated with echocardiographic parameters reflecting AS severity and a specific pattern of valvular calcification in severe AS.

## Methods

### Patients

**Fig. 1:**
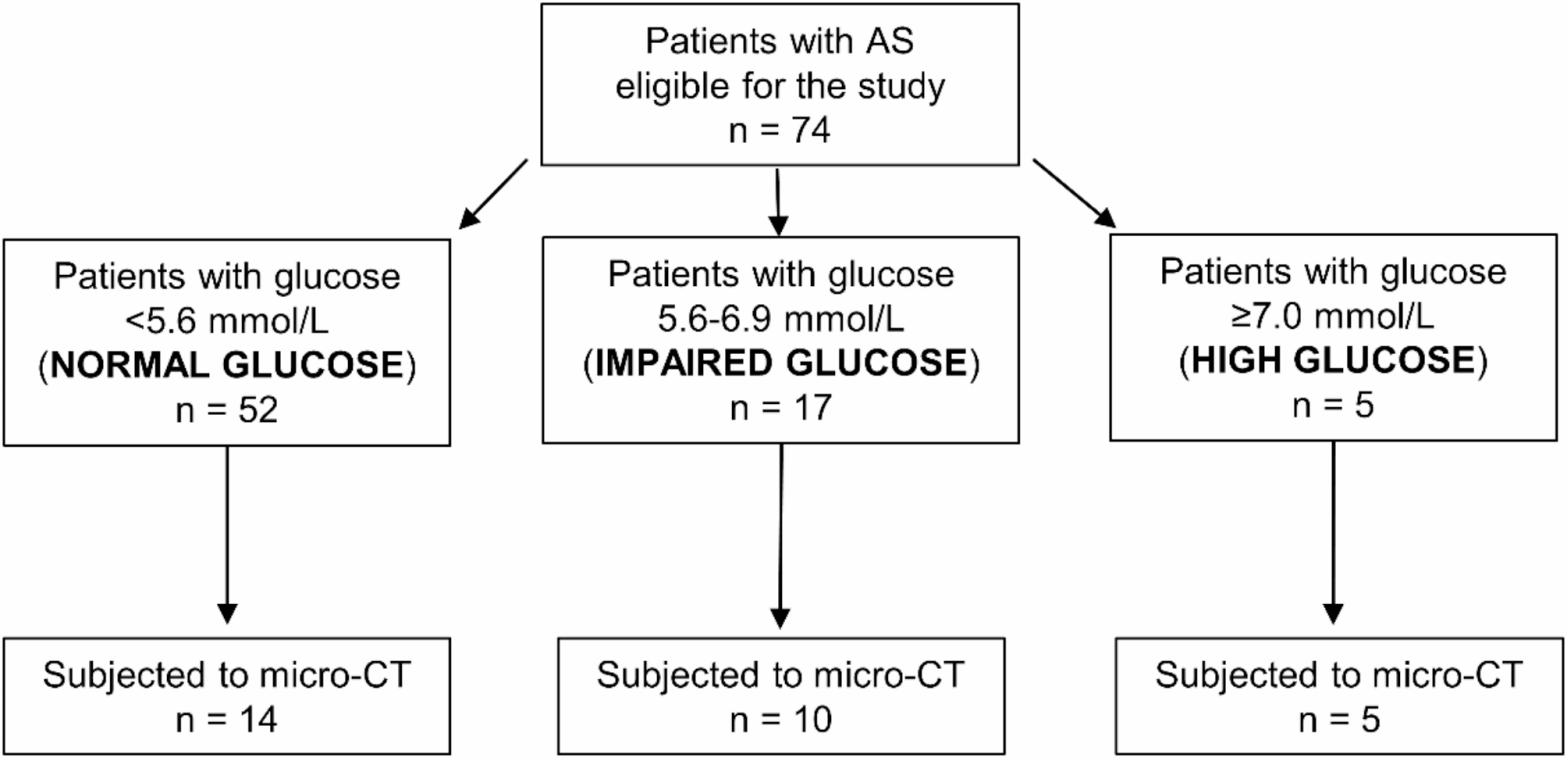
Study flow chart

In this study, 29 patients with severe symptomatic AS, who underwent their first elective SAVR, were recruited between 2020 and 2024 at the Department of Cardiovascular Surgery and Transplantology, St. John Paul II Hospital, Krakow, Poland. All patients with isolated severe AS were consecutively enrolled in the study to minimize the risk of selection bias (Fig. 1). Exclusion criteria were as follows: previously diagnosed type 2 DM [14], chronic kidney disease stages 4 and 5 or dialysis-dependent patients, left ventricular ejection fraction (LVEF) of < 40%, atherosclerotic vascular disease requiring revascularization, clinically evident coronary artery disease on coronary angiography and peripheral artery disease on angiography, documented myocardial infraction or stroke, history of chronic obstructive pulmonary disease, autoimmune disease or cancer, bicuspid aortic valve, any significant valvular disease such as rheumatic disease, mitral or aortic regurgitation.

Medical history, current treatment, and demographic information were gathered through a standardized questionnaire. Severe AS was defined by the following criteria on transthoracic echocardiography: mean transvalvular pressure gradient (PG_mean_) ≥ 40 mm Hg, peak transvalvular velocity (V_max_) ≥ 4.0 m/s, and aortic valve area (AVA) ≤ 1 cm^2^ [15]. No individuals with low-flow low-gradient AS were recruited. All echocardiographic measurements were performed by an experienced cardiologist using a Toshiba APLIO 80 (Toshiba, Tokyo, Japan). All patients with AS had a tricuspid aortic valve confirmed during the SAVR procedure by a cardiac surgeon.

According to the 2024 American Diabetes Association (ADA) criteria [14], normoglycaemia was defined as a fasting glucose level of < 5.6 mmol/L, impaired glucose as fasting glucose levels of 5.6–6.9 mmol/L or HbA_1c_ levels of 5.7–6.4%, and high glucose as fasting glucose levels of ≥ 7.0 mmol/L. We included subjects confirmed to be fasting overnight prior to blood sampling, and with measured glucose prior to SAVR. None of the patients included in the study were treated with antidiabetic agents such as metformin, insulin, sodium-glucose cotransporter-2 inhibitors, or glucagon-like peptide-1 agonists.

Arterial hypertension was identified based on a history of elevated blood pressure (systolic > 140 mm Hg or diastolic > 90 mm Hg) or prior use of antihypertensives. Hypercholesterolemia was diagnosed either by a total cholesterol level ≥ 5.0 mmol/L, documented in medical records, or through the use of cholesterol-lowering treatments. Atherosclerosis was diagnosed based on angiographic documentation of coronary artery stenosis greater than 20% in diameter, and such patients were excluded from the study to eliminate the potential influence of nonobstructive atherosclerosis [16].

The Ethical Committee (Krakow Medical District Chamber and Jagiellonian Univeristy Bioethics Committee, Poland, approval numbers: 8/KBL/OIL/2019 and 1072.6120.81.2024) approved the study and all participants provided written informed consent in accordance with the Declaration of Helsinki.

### Laboratory investigations

Fasting venous blood samples were collected from the antecubital vein of all patients with AS prior to undergoing SAVR. Citrated blood (with a 9:1 ratio of 0.106 M sodium citrate) was centrifuged at 2500 g for 20 min at 20 °C, while blood collected in EDTA or serum tubes was centrifuged at 1600 g for 10 min at 4 °C and stored at − 80 °C for later analysis. Routine laboratory assays were employed to measure glucose, creatinine, lipid profile, and C-reactive protein (CRP). HbA_1c_ was determined using turbidimetric inhibition immunoassay TINIA (Roche Diagnostics, Mannheim, Germany). Fibrinogen levels were determined using the von Clauss method (Instrumentation Laboratory, Bedford, MA, USA).

### ELISA testing

Human AGEs (all species with the predominance of N(6)-Carboxymethyllysine) and circulating human soluble receptor for AGEs (sRAGE) concentrations in EDTA plasma samples were assayed quantitatively using commercial ELISA kits (EIAab, Wuhan, China and Boster Biological Technology, California, USA, respectively) in accordance with the manufacturer’s instructions.

### Preparation of the aortic valves

Aortic valves were obtained during the SAVR procedure (74% via full median sternotomy and 26% using upper hemisternotomy), carefully removed to preserve the integrity of the individual cusps, and promptly transported to the laboratory. The leaflets were rinsed three times with phosphate-buffered saline (PBS, without calcium and magnesium; Biowest, Nuaillé, France). The non-coronary leaflet was preserved in 10% formalin and used for micro-CT analysis (Sigma-Aldrich, St. Louis, MO, USA), while the remaining two were stored for subsequent analysis.

### Micro-computed tomography (micro-CT)

Micro-CT measurements were performed for the quantitative and qualitative characterization of valvular calcification. Samples were analysed using a Nanotom 180 S tomograph (GE Sensing & Inspection Technologies, Billerica, MA, USA). To prevent tissue dehydration, the samples were enclosed in an airtight plastic container with water at the bottom. During the examination, the sample was placed on an internal table above the water level and scanned at a source voltage of 70 kV and an X-ray tube current of 100 µA. No filters were applied. During a full rotation of the sample, 2,100 projections were recorded, with each projection averaged from three exposures. The duration of a single exposure was 500 ms. Images were captured using a Hamamatsu detector with a resolution of 2304 × 2304 pixels, and the image magnification was set to 5, resulting in a voxel size of 10 μm. All data were recorded at 32-bit resolution. The following parameters were evaluated:

- Calcium volume (CV): represents total calcium volume,
- Surface volume (SV): indicates the volume of surface layer of the calcification, it is proportional to surface of the calcification,
- Calcium volume/surface volume (CV/SV) ratio: degree of corrugation of calcified parts in aortic valve,
- And trabecular thickness (TbTh): measures the distribution of mean, maximal and deviation size of individual calcific nodules.

Valvular leaflet calcification expressed as a ratio of total calcium area and total valve area was calculated using the ImageJ software.

Reconstruction of the scanned samples was performed using GE software datos|x version 2.1.0, employing the Feldkamp algorithm for cone beam X-ray CT [17]. Post-reconstruction data were processed using VGStudio Max 3.1 software, as well in Fiji software. During post-processing, the plastic container was digitally removed. All samples were subjected to the same image processing procedure. Initially, images were slightly denoised using a median filter with a radius of 10 μm. Subsequently, the sample was geometrically separated from the table and the background (the air surrounding the sample) was removed through thresholding. The investigators performing micro-CT procedures was blinded to patients’ history.

### Statistical analysis

Categorical variables are presented as numbers and percentages, while continuous variables are presented as mean ± standard deviation (SD) or median and interquartile range [Q1–Q3]. Categorical variables were analysed using two-tailed Fisher’s exact test. Normality was analysed by the Shapiro–Wilk test. Differences between the groups were compared using the Student’s t-test or Mann–Whitney U test, as appropriate. Correlations between continuous variables were calculated using Spearman’s correlation coefficients (sample size < 30). To compare continuous variables between multiple groups, analysis of variance (ANOVA) with Tukey–Kramer HSD post-hoc analysis (for unequal group sizes) or the Kruskal–Wallis test was performed. A *p*-value < 0.05 was considered statistically significant.

The univariable linear regression models were performed to identify associations between micro-CT parameters and demographic, clinical, and laboratory variables. Variables that were associated with CV or TbTh_mean_ with a significance level of *p*-value < 0.2 in the univariable models were selected and the multivariable linear models were adjusted for sex and BMI. Standard error of the coefficient (SE β) denotes the uncertainty in the estimated regression coefficient β. Based on available data on associations between micro-CT and echocardiographic parameters in mild-to-moderate patients with AS sample size was calculated [18]. At least 4 patients per group were required to achieve 90% statistical power with a *p*-value of 0.05. Statistical analysis was performed using STATISTICA software (Version 13.3, TIBCO Software, Palo Alto, CA, USA).

## Results

Fourteen (48%) of the studied patients with AS had fasting plasma glucose within the reference range (< 5.6 mmol/L), indicating normoglycaemia, and 15 (52%) had elevated plasma glucose levels, including 10 (67%; 34.5% of all) patients with impaired fasting glucose in the range of 5.6–6.9 mmol/L, and 5 (33%; 17.2% of all) with high glucose ranging from 7 to 7.6 mmol/L, however, with HbA_1c_ ≤ 6.5% (Fig. 1). Patients with normoglycaemia and those with impaired and high glucose did not differ in demographic characteristics, risk factors, or medication use, except for a lower frequency of angiotensin converting enzyme (ACE) inhibitors use in the former group (Table 1). There were 6 individuals with LVEF < 50% (4 patients with normal plasma glucose and 2 patients with impaired fasting glucose; range: 40–48%, median: 40%, Q1–Q3:40–44%), all of whom met the criteria for classical high-gradient AS [14]. Of note, a total of 13 patients fulfilled the criteria for metabolic syndrome [19]. This condition was present in 10 individuals with impaired and high fasting glucose (characterized by BMI ≥ 30 kg/m^2^ and hypertension, nine of them on statin therapy), and in three patients with normal glucose, who met the definition based on BMI ≥ 30 kg/m^2^, hypertension, and statin use. Patients with impaired and high plasma glucose had significantly higher HbA_1c_ and AGEs levels (6.2 ± 0.3% vs. 5.3 ± 0.3%, *p* < 0.0001 and 10.7 [9.7–11.8] ng/ml vs. 7.4 [6.4–7.8] ng/ml, *p* = 0.00011, respectively), but not sRAGE compared to those with normal glucose levels (Table 1). No intergroup differences were found in other routine laboratory parameters (Table 1). Furthermore, individuals with impaired and high plasma glucose had 19.2% higher PG_max_ (87 [77–93] mm Hg versus 73 [68–79] mm Hg, *p* = 0.009) and 9.3% higher V_max_ (4.7 [4.4–4.8] m/s vs. 4.3 [4.1–4.4] m/s, *p* = 0.009) compared to those with normoglycaemia (Table 1).

**Table 1:** Baseline characteristics of the patients with aortic stenosis stratified according to plasma glucose levels

Variable	Normal fasting glucose (*n* = 14)	Impaired and high fasting glucose (*n* = 15)	*p*-value
Age, years	72 [65–73]	72 [70–76]	0.24
Male, n (%)	8 (57.1)	12 (80)	0.25
BMI, kg m^− 2^	30.4 ± 4.8	31.1 ± 3.9	0.67
Risk factors, n (%)			
Obesity (BMI ≥ 30 kg m^− 2^)	6 (42.9)	10 (66.7)	0.27
Arterial hypertension	13 (92.9)	15 (100)	0.48
Hypercholesterolemia	11 (78.6)	14 (93.3)	0.33
eGFR < 60 mL/min/1.73 m^2 a^	4 (28.6)	6 (40)	0.70
LDL-cholesterol < 2.5 mmol/L^b^	8 (57.1)	11 (73.3)	0.45
LDL-cholesterol < 1.8 mmol/L^c^	3 (21.4)	5 (33.3)	0.68
Current smoking	1 (7.1)	1 (6.7)	0.99
Medications, n (%)			
Beta-blockers	13 (92.9)	15 (100)	0.48
Acetylsalicylic acid	8 (57.1)	6 (40)	0.47
ACE inhibitors	8 (57.1)	14 (93.3)	0.035
Statins	10 (71.4)	14 (93.3)	0.17
Echocardiographic parameters			
PG_mean_, mm Hg	44 [42–52]	50 [46–64]	0.052
PG_max_, mm Hg	73 [68–79]	87 [77–93]	0.009
V_max_, m/s	4.3 [4.1–4.4]	4.7 [4.4–4.8]	0.009
AVA, cm^2^	0.83 ± 0.17	0.75 ± 0.12	0.17
LVEF, %	54 ± 9	55 ± 8	0.64
Laboratory investigations			
Glucose, mmol/L	5.3 [4.9–5.5]	6.3 [6.0–7.1]	< 0.0001
HbA_1c_, %	5.3 ± 0.3	6.2 ± 0.3	< 0.0001
Fibrinogen, g/L	3.4 ± 0.5	3.7 ± 0.6	0.22
Creatinine, µmol/L	88 ± 19	102 ± 27	0.13
CRP, mg/L	1.8 [1.0–4.2]	1.6 [1.1–5.9]	0.71
Total cholesterol mmol/L	4.2 ± 1.3	3.6 ± 0.9	0.19
LDL-cholesterol, mmol/L	2.5 [2.1–3.4]	2.2 [1.6–2.7]	0.35
HDL-cholesterol, mmol/L	1.2 [1.2–1.6]	1.1 [0.9–1.4]	0.1
Triglycerides, mmol/L	1.1 [0.8–1.6]	1.1 [0.9–1.5]	0.87
Plasma markers			
AGEs, ng/mL	7.4 [6.4–7.8]	10.7 [9.7–11.8]	0.00011
sRAGE, pg/mL	1512 ± 457	1730 ± 714	0.34

Data presented as numbers (percentages), mean ± SD or medians [interquartile range]. *P*-values of < 0.05 were considered statistically significant

*ACE inhibitors * angiotensin converting enzyme inhibitors,* AGEs* advanced glycation end products,* AS* aortic stenosis,* AVA* aortic valve area,* CRP* C-reactive protein, *HbA*_*1c*_ glycated haemoglobin,* LVEF* left ventricular ejection fraction, *PG*_*mean*_ mean transvalvular pressure gradient, *PG*_*max*_ maximal transvalvular pressure gradient, *sRAGE* soluble receptor for advanced glycation end products,* V*_*max*_ peak transvalvular velocity

^a^Patients with eGFR < 60 mL/min/1.73 m^2^, none on dialysis

^b^For patients with intermediate cardiovascular risk

^c^For patients at high cardiovascular risk

**Fig. 2:**
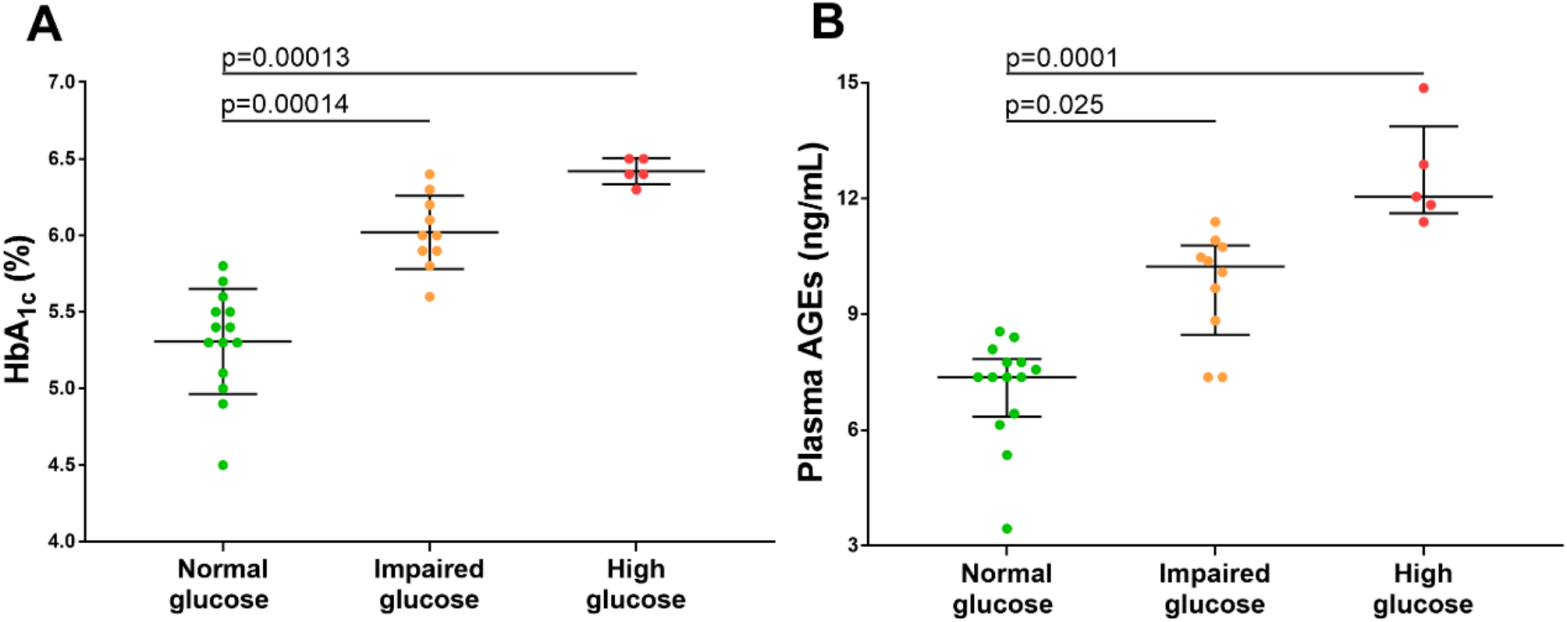
HbA_1c_ and plasma AGEs concentrations in patients with aortic stenosis, stratified by glucose level. Dot-plots comparing HbA_1c_ levels (panel **A**) and AGEs concentrations (panels **B**) in patients with AS divided according to ADA classification [14]: normal glucose level: < 5.6 mmol/L (green circles, *n* = 14), impaired fasting glucose: 5.6–6.9 mmol/L (orange circles, *n* = 10) and high fasting glucose: ≥7.0 mmol/L (red circles, *n* = 5). HbA_1c_ levels are presented as mean ± SD, while AGEs concentrations are presented as medians [interquartile range]. ANOVA or the Kruskal-Wallis test was performed, depending on the data distribution. Abbreviations as in Table 1

After stratifying patients with AS into three groups based on plasma glucose levels (< 5.6 mmol/L, ≥ 5.6–6.9 mmol/L, and 7.0–7.6 mmol/L), we observed differences in HbA_1c_ and AGEs levels. Patients with glucose levels in the ranges of 5.6–6.9 mmol/L and 7.0–7.6 mmol/L exhibited higher levels of HbA_1c_ by 13.2% (6.0 ± 0.2% vs. 5.3 ± 0.3%, *p* = 0.00014) and 20.8% (6.4 ± 0.1% vs. 5.3 ± 0.3%, *p* = 0.00013), respectively, compared to those with normal glucose levels (Fig. 2A). Similarly, AGEs concentrations were significantly elevated by 37.8% and 62.2% in individuals with glucose concentrations between 5.6 and 6.9 mmol/L (10.2 [8.8–10.7] ng/ml vs. 7.4 [6.4–7.8] ng/ml, *p* = 0.025) and 7.0−7.6 mmol/L (12 [11.8–12.9] ng/ml vs. 7.4 [6.4–7.8] ng/ml, *p* = 0.00012), respectively, compared to those with normoglycaemia (Fig. 2B). No significant differences were observed in plasma sRAGE levels, routine laboratory parameters, including creatinine, LDL-C, and CRP, demographic characteristics, risk factors, or medication use among these three groups (all *p* > 0.05).

Positive associations were found between plasma glucose levels and HbA_1c_ (*r* = 0.79, *p* < 0.0001) as well as plasma AGEs (*r* = 0.74, *p* < 0.0001), but not with sRAGE (*p* > 0.05). A moderate positive correlation was observed between plasma AGEs and sRAGE (*r* = 0.53, *p* = 0.003). Importantly, plasma glucose levels correlated with disease severity, as reflected by both transvalvular pressure gradients (PG_mean_: *r* = 0.39, *p* = 0.035 and PG_max_: *r* = 0.44, *p* = 0.016), V_max_ (*r* = 0.44, *p* = 0.016) and AVA (*r* = − 0.45, *p* = 0.014). Similarly, plasma concentrations of AGEs were associated with PG_max_ (*r* = 0.44, *p* = 0.016), V_max_ (*r* = 0.44, *p* = 0.016) and AVA (*r* = − 0.42, *p* = 0.025). No associations were observed between HbA_1c_ or sRAGE and echocardiographic parameters, even after stratifying patients into impaired and high plasma glucose and normoglycaemic groups.

### Micro-CT

**Fig. 3:**
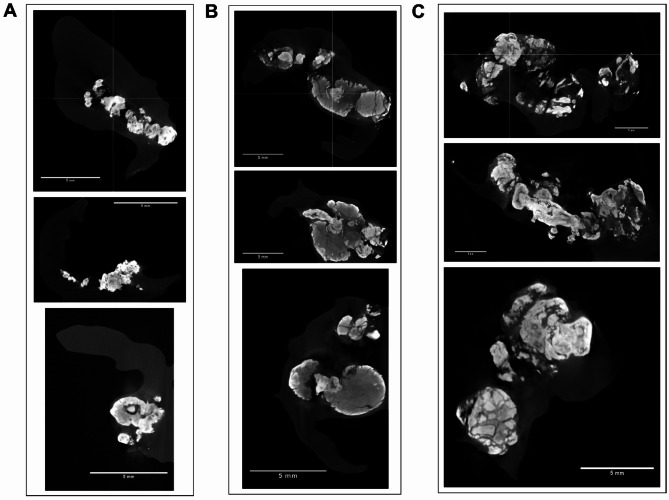
Representative cross-sections of the valve leaflets in normoglycaemic patients with AS and those with impaired or high glucose levels acquired via micro-CT. Cross-sections (XY, XZ, and YZ plane) of the valve leaflets, demonstrating typical valvular features obtained using micro-CT, with regard to glycaemic status;** A** a normoglycaemic patient with AS,** B** impaired, and** C** high plasma glucose in patients with AS. Scale bar, 5 mm. Valvular leaflet calcification expressed as a ratio of total calcification area and total leaflet area was calculated for each leaflet

**Fig. 4:**
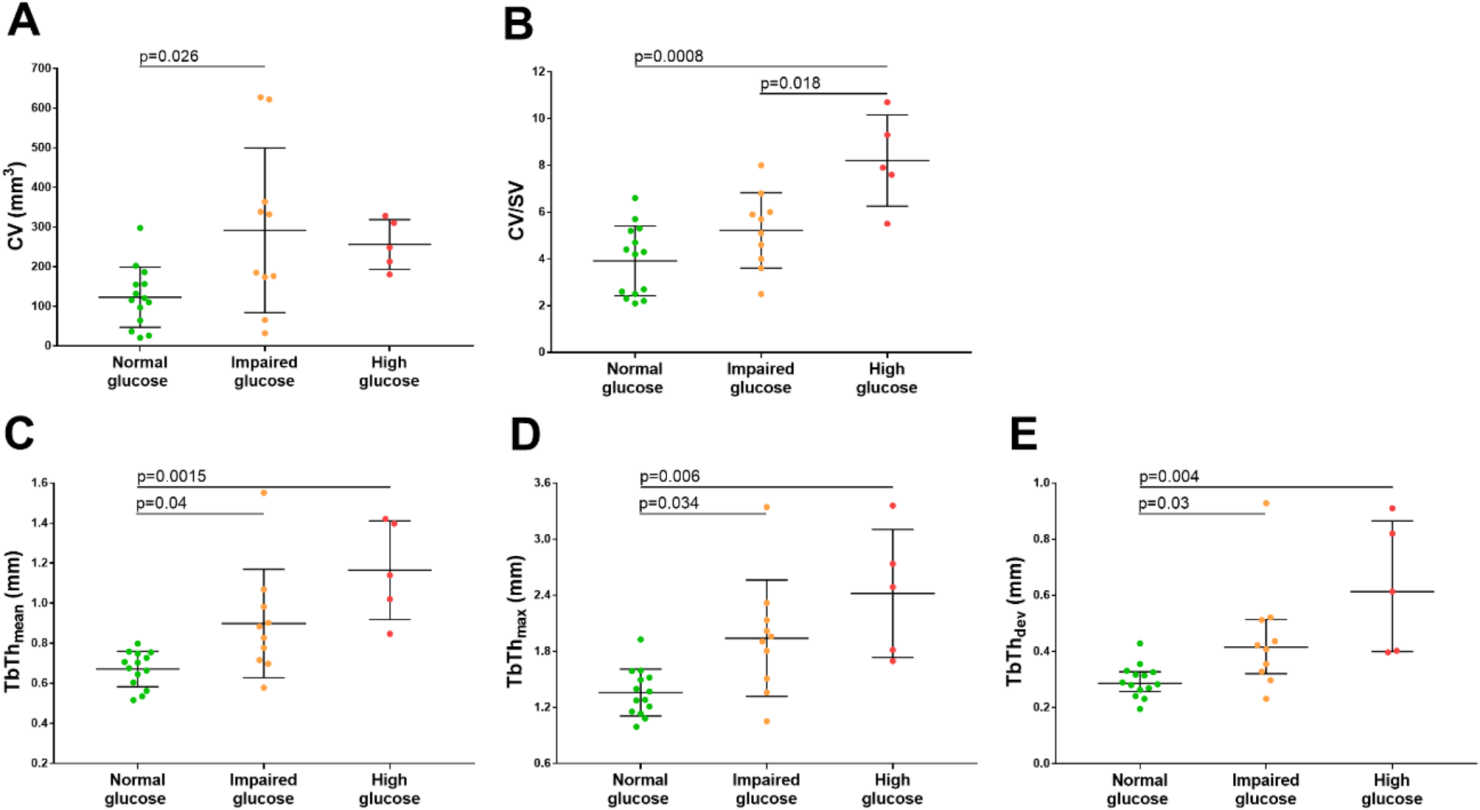
Micro-CT analysis of valvular calcification in patients with AS, stratified by glucose level. Dot-plots comparing micro-CT parameters (panels **A–E**) in patients with AS divided according to ADA classification [14]: normal glucose level: <5.6 mmol/L (green circles, *n* = 14), impaired fasting glucose: 5.6–6.9 mmol/L (orange circles, *n* = 10) and high fasting glucose: ≥7.0 mmol/L (red circles, *n* = 5). TbTh_dev_ is presented as medians [interquartile range], while CV, CV/SV ratio, TbTh_mean_, and TbTh_max_ are presented as mean ± SD. ANOVA or the Kruskal-Wallis test was performed, depending on the data distribution. Abbreviations as in Table 2

Total valvular leaflet calcification accounted for 16.1 ± 9.2% in normoglycemic patients, 35.4 ± 10.2% in patients with impaired glucose, and 47.3 ± 11.4% in patients with high glucose (*p* < 0.001). In patients with AS and impaired and high plasma glucose compared to the remainder we observed a 127.6% increase in mean CV (279.9 ± 171 mm^3^ vs. 123.0 ± 76 mm, *p* = 0.004) and 59% higher mean CV/SV ratio (6.2 ± 2.2 vs. 3.9 ± 1.5, *p* = 0.003), but not SV (*p* > 0.05, Table 2). Also calcium deposits microarchitecture parameters, namely TbTh_mean_ (0.99 ± 0.3 mm vs. 0.67 ± 0.1 mm, *p* = 0.0005), TbTh_max_ (2.09 ± 0.66 mm vs. 1.36 ± 0.25 mm, *p* = 0.0005), and TbTh_dev_ (0.42 [0.36–0.61] mm vs. 0.29 [0.26–0.33] mm, *p* = 0.0007) were increased byabout 50% in subjects with impaired and high plasma glucose compared to those with normoglycaemia (Table 2). Furthermore, we observed differences in structural characteristics between the groups: in the impaired and high glucose group, the deposits were generally larger and more mineralized, forming more corrugated clusters (Fig. 3B and C), whereas in the normoglycaemic group, the deposits were smaller in terms of calcium volume, but also less folded and calcified (Fig. 3A). Interestingly, the highest CV value was observed in patients with glucose levels of 5.6–6.9 mmol/L, showing an increase of 137.4% compared to those with normal glucose levels (292 ± 208 mm^3^ vs. 123 ± 76 mm^3^, *p* = 0.026) (Fig. 4A). Patients with glucose levels between 7.0 and 7.6 mmol/L demonstrated elevated CV/SV ratio, which was 110.3% and 57.7% higher than in individuals with normal glucose levels (8.2 ± 1.9 vs. 3.9 ± 1.5, *p* = 0.0008) and those with glucose levels in the 5.6–6.9 mmol/L range (8.2 ± 1.9 vs. 5.2 ± 1.6, *p* = 0.018), respectively (Fig. 4B). Furthermore, patients with glucose levels of 5.6–6.9 mmol/L had 32.8% increased TbTh_mean_ (0.89 ± 0.27 mm vs. 0.67 ± 0.1 mm, *p* = 0.04) and those with glucose between 7.0 and 7.6 mmol/L had 73.1% increased TbTh_mean_ (1.16 ± 0.25 mm vs. 0.67 ± 0.1 mm, *p* = 0.0015) compared to those with normal glucose levels (Fig. 4C). Similarly, patients with glucose between 5.6 and 6.9 mmol/L had about 40% increased TbTh_max_ (1.94 ± 0.62 mm vs. 1.36 ± 0.25 mm, *p* = 0.034) and TbTh_dev_ (0.41 [0.33–0.51] mm vs. 0.29 [0.26–0.33] mm, *p* = 0.03) compared to those with normoglycaemia, while those with glucose 7.0–7.6 mmol/L had 77.9% higher TbTh_max_ (2.42 ± 0.68 mm vs. 1.36 ± 0.25 mm, *p* = 0.006) and 110.3% higher TbTh_dev_ (0.61 [0.40–0.82] mm vs. 0.29 [0.26–0.33] mm, *p* = 0.004) compared to normal glucose (Fig. 4D and E).

**Table 2:** Micro-CT parameters of valvular leaflets obtained from patients with aortic stenosis divided according to glucose level

Variable	Normal fasting glucose (*n* = 14)	Impaired and high fasting glucose (*n* = 15)	*p*-value
CV, mm^3^	123.0 ± 76	279.9 ± 171	0.004
SV, mm^3^	26.3 [20.7–42.6]	35.5 [23.2–55.9]	0.11
CV/SV	3.9 ± 1.5	6.2 ± 2.2	0.003
TbTh_mean_, mm	0.67 ± 0.1	0.99 ± 0.3	0.0005
TbTh_max_, mm	1.36 ± 0.25	2.09 ± 0.66	0.0005
TbTh_dev_, mm	0.29 [0.26–0.33]	0.42 [0.36–0.61]	0.0007

Data presented as mean ± SD or median [interquartile range]. *P*-values of < 0.05 were considered statistically significant

*CV* calcium volume,* CV/SV ratio* calcium volume/ surface volume ratio,* SV* surface volume,* TbTh*_*max*_ maximal trabecular thickness,* TbTh*_*mean*_ mean trabecular thickness,* TbTh*_*dev*_ deviation of trabecular thickness

**Fig. 5:**
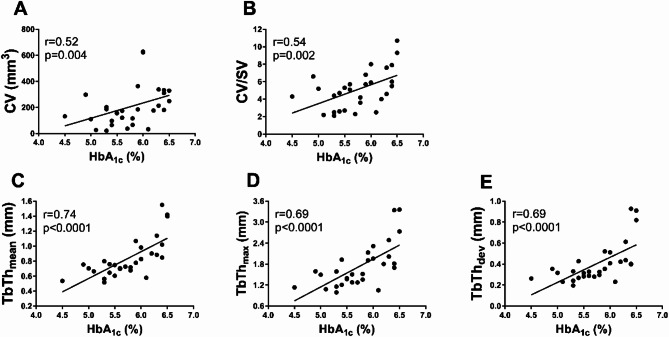
Associations between HbA_1c_ levels and micro-CT parameters. The scatterplots show positive correlations between glycated haemoglobin (HbA_1c_) levels and micro-CT parameters, such as **A** CV, **B** CV/SV ratio, **C** TbTh_mean_, **D** TbTh_max_, and **E** TbTh_dev_. Abbreviations as in Table 2

**Fig. 6:**
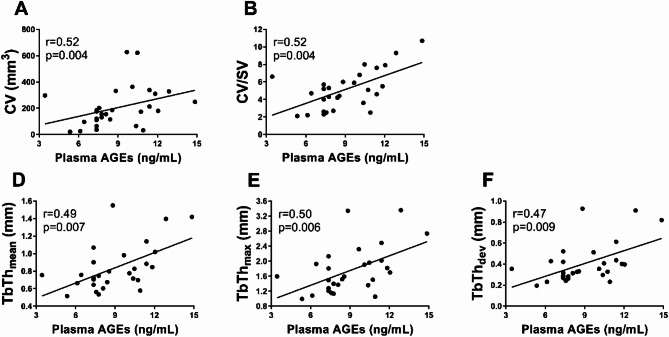
Associations between plasma concentrations of AGEs and micro-CT parameters. The scatterplots show positive correlations between advanced glycation end products (AGEs) levels and micro-CT parameters, namely **A** CV, **B** CV/SV ratio, **C** TbTh_mean_, **D** TbTh_max_, and **E** TbTh_dev_. Abbreviations as in Table 2

Plasma glucose positively correlated with all micro-CT parameters, including CV (*r* = 0.67, *p* < 0.001), SV (*r* = 0.38, *p* = 0.04), and CV/SV ratio (*r* = 0.69, *p* < 0.0001), as well as with TbTh_mean_ (*r* = 0.69, *p* < 0.0001), TbTh_max_ (*r* = 0.68, *p* < 0.0001), and TbTh_dev_ (*r* = 0.67, *p* < 0.0001), even after adjustment for total cholesterol (all *p* < 0.05). We also observed associationsof HbA_1c_ with micro-CT indices (Fig. 5A–E). Similarly, plasma concentrations of AGEs were associated with micro-CT parameters (Fig. 6A-E), except for SV (*p* > 0.05). No associations were observed between plasma sRAGE levels and micro-CT measures, even after stratifying patients into those with normoglycaemia and those with impaired fasting glucose (all *p* > 0.05).

Micro-CT indices of valvular calcification, namely the CV/SV ratio TbTh_mean_, TbTh_max_ and TbTh_dev_, were also associated with PG_mean_, PG_max_, V_max_ and AVA (Table 3). No associations were found between micro-CT indices and age, BMI, arterial hypertension, or other routine laboratory parameters (all *p* > 0.05). There were no differences in micro-CT parameters between patients with normal and reduced LVEF (all *p* > 0.05).

**Table 3:** Associations between echocardiographic measures and micro-CT parameters

Variable	*r*	*p*-value
PG_mean_		
CV/SV	0.53	0.004
TbTh_mean_	0.44	0.019
TbTh_max_	0.40	0.008
TbTh_dev_	0.40	0.01
PG_max_		
CV/SV	0.50	0.006
TbTh_max_	0.41	0.032
V_max_		
CV/SV	0.50	0.006
TbTh_max_	0.41	0.032
AVA		
CV/SV	− 0.59	0.001
TbTh_mean_	− 0.45	0.014
TbTh_max_	− 0.38	0.045
TbTh_dev_	− 0.43	0.02

*P*-values of < 0.05 were considered statistically significant

Abbreviations as in Tables 1 and 2

Linear regression analysis revealed that higher CV as well as TbTh_mean_ were associated with higher glucose (per 1 mmol/L increase; β = 0.51, standard error (SE) β = 0.17, 95% confidence interval (CI) 0.17–0.85 and β = 0.74, SE β = 0.13, 95% CI 0.47–1.01; Supplemental Table 1).

## Discussion

Our study demonstrated for the first time that in patients with severe AS, the pattern of valvular calcification is associated with both short- and long-term glycaemic control parameters, namely glucose and HbA_1c_ levels as well as with AGEs concentration. Patients with impaired or high plasma glucose had denser calcium deposits with a higher total volume, which was associated with higher transvalvular pressure gradients and V_max_. The present findings suggest that impaired fasting glucose may actively contribute to progressive changes in the architecture of aortic valve leaflets, characterized by increased calcium volume, degree of corrugation, and enhanced structural integrity, as evidenced by greater trabecular thickness. Moreover, we provide a more detailed characterization of valvular calcification through ex vivo micro-CT analysis, which represents the novelty of this research. This advanced imaging technique allows for a more detailed and precise assessment of the calcification process, providing insights that go beyond traditional methods.

Such modalities like, for instance, CT angiography (CTA) are unable to provide images of quality comparable to micro-CT. In a study analysing characteristics of ultra-high resolution CTA with photon-counting detectors, the maximal acquired resolution was 0.11 × 0.11  mm^2^ and 0.16 mm in-plane and through-plane, respectively [20].Meanwhile, voxel size in images obtained in our study reached 0.01 mm, allowing for capturing of fine details and observation of subtleties in internal structure of analysed calcific nodules. In comparison, other studies that used previously mentioned CTA modalities for evaluation of calcification in cardiovascular system utilized imaging devices allowing for 0.2 mm slices [21, 22]. Therefore, currently used CTA techniques, including modalities supported by artificial intelligence [23], would not be suitable for observation of TbTh values of order of magnitude given in this study. Ex vivo measurements allow for precise assessment of calcium deposits on valve leaflets without interference from confounding factors [18]. Thanks to that particular characteristic, we were able to observe associations of echocardiographic parameters with not only calcific tissue content, but also TbTh variables, suggesting that larger calcific nodules observed in patients with impaired fasting glucose may contribute to severity of AS. This observation shows that future AS risk assessment with more precise modalities that would allow for in vivo imaging should take into consideration distribution of calcific tissue. Moreover, our report may suggests that in patients with impaired fasting glucose and AS, calcium scoring CT should be performed more often to evaluate the Agatston score to monitor valvular mineralization.

A large body of evidence shows that DM and metabolic syndrome are associated with increased aortic valve calcification and the acceleration of AS progression [6, 9, 24–28]. A recent study by Wang et al. [28] showed using CT in a large cohort of 30,154 middle-aged individuals (50 to 64 years), however without AS, that pre-diabetes, newly detected DM and DM, were independently associated with aortic valve calcification (OR 1.16 [95% CI 1.02–1.31]; OR 1.34 [95% CI 1.05–1.71]; OR 1.61 [95% CI 1.34–1.93], respectively). The present study extended the observations by Wang et al. [28] and showed complete quantitative characterization of valvular calcification using ex vivo micro-CT in both patients with AS and normoglycaemia and those with impaired and high fasting glucose. In our observational analysis, the calcification volume, folding, and trabecular density within calcium deposits were higher in patients with increased glucose levels and the highest HbA_1c_ compared to those with normal glucose levels. Furthermore, given the similarities between AS and atherosclerosis, this study is in line with the findings of Cho et al. [29], who observed that persistent pre-diabetes was associated with a higher prevalence of coronary artery calcification via CT scans (OR 1.13 [95% CI 1.08–1.18]) in a large cohort study, involving young and middle-aged Korean participants. It deserves attention that plasma glucose is a highly variable parameter. Previous research often demonstrates that single-point glucose levels, such as fasting glucose, correlate with inflammatory markers like CRP and interleukin-6 [30–32]. This provides a scientific basis for using single measurements as a proxy for glucose’s impact on inflammation, which is the key factor driving development and AS progression. Interestingly, evidence from a recent large-scale Swedish cohort study involving over 324,000 participants demonstrated that even a single baseline glucose measurement could predict an increased risk of AS [33]. Adjusted hazard ratios (HRs) showed a stepwise increase in AS risk: 1.36 (95% CI 1.24–1.50) for impaired fasting glucose, 1.79 (95% CI 1.60–1.99) for elevated glucose, and 2.21 (95% CI 1.80–2.73) for DM over a mean follow-up of 25.9 years [33]. This supports the notion that even short-term glycaemic status may reflect long-term cardiovascular risk. On the other hand, HbA_1c_ reflects average plasma glucose levels over the past 3 months. Therefore, in our study, we measured both plasma glucose and HbA_1c_ in a fasting state, prior to the SAVR procedure. We also incorporated plasma AGEs levels into our analysis, as AGEs have been strongly associated with long-term diabetes complications. Moreover, the strong correlation with HbA_1c_ and plasma AGEs concentrations supports the appropriateness of the categories we applied, indicating that the observed impaired plasma glucose reflects true glycaemic disturbances in accordance with the ADA guidelines [14]. Therefore, the analysis included plasma AGEs and sRAGE levels, providing new evidence for glycation as a factor associated with aortic valve calcification.

AGEs regulate various cellular processes by cross-linking both intracellular and extracellular matrix proteins [34] or by interacting with RAGE on the cell surface, leading to increased production of inflammatory molecules [35]. This excessive cross-linking, particularly affecting collagen, laminin, vitronectin, and elastin [36, 37], disrupts the natural flexibility of these matrix proteins, rendering them rigid and impairing their functional properties. Notably, collagen, as a major structural protein, is particularly prone to glycation due to its long half-life of approximately 10 years. Furthermore, research suggests that collagen glycation enhances myofibroblast formation and migration, playing a role in fibrosis development in diabetes [38], while glycated collagen has also been shown to impair endothelial cell function and may be a key factor in the progression of atherosclerotic plaque formation in this condition [39]. It has also been demonstrated that AGEs enhanced glycation of valvular proteins within stenotic leaflets [8] and induced calcification, as evidenced by increased expression of typical bone proteins, including osteocalcin, osteopontin and alkaline phosphatase, in human aortic smooth muscle cells [40] and rat vascular smooth muscle cells [41]. Importantly, the current study demonstrated correlations not only between glucose or HbA_1c_ levels and micro-CT findings or echocardiographic results but also with plasma AGEs concentrations, demonstrating an association between glycation and valve calcification. AGEs are known to accumulate in tissues and are associated with the progression of pre-diabetes and diabetes [42]. This study supports evidence from our previous observations [8], where valvular AGEs expression correlated with clinical measures of AS severity, such as PG_mean_ and AVA. Given that plasma AGEs levels were also associated with AVA, it becomes evident that AGEs have a multifaceted relationship with AS severity in DM, even at the advanced stage of the disease, where surgical intervention is unavoidable [8]. Moreover, diabetic patients with HbA_1c_ > 7.0% were characterized by higher PG_mean_ and PG_max_ compared to those with HbA_1c_ ≤ 7.0% [8]. We also showed that in diabetic patients, valvular expression of BMP-2 correlated with HbA_1c_ and fructosamine levels [9]. These findings underscore the importance of glycaemic control in the progression of AS and suggest that monitoring and managing plasma glucose levels could be beneficial in mitigating the severity of AS. Interestingly, in contrast to earlier findings, we did not observe any associations between plasma sRAGE levels and echocardiographic measures [8]. This discrepancy may stem from the fact that the present study excluded patients with DM. In contrast, the previous study focused on patients with both well- and poorly-controlled DM type 2 [8]. It has been demonstrated that exposure to increased plasma glucose levels rapidly accelerates the formation of AGEs, and their subsequent accumulation within the aortic leaflets triggers osteoblastic differentiation of VICs [10, 43]. Hyperglycaemia-induced AGEs formation enhances oxidative stress, which further activates the NF-κB signalling pathway [44]. This leads to the up-regulation of pro-inflammatory cytokines and key pro-calcification factors, such as BMP-2,-4, osteopontin, osteocalcin, runt-related transcription factor 2 (Runx-2) and Smad1/5/8, contributing to increased calcium deposition within the valves [9, 45]. The resulting VICs activation, driven by the NF-κB pathway, leads to the remodelling and dysfunction of the aortic valve, marked by increased matrix metalloproteinaseproduction, extracellular matrix protein synthesis, and up-regulated expression of cell adhesion molecules, integrins, and pro-inflammatory cytokines [45, 46], contributing to the faster disease progression in individuals with DM. Therefore, elevated glucose levels may contribute to the development and/or progression of AS, potentially even at prediabetic levels before DM is diagnosed. However, further research is needed to better understand the role of glycaemic control in modulating AS progression. Moreover, high LDL-C and particular lipoproteins are linked to increased AS risk [47, 48], while statin therapy has not proven effective [49]. The SEAS study found that simvastatin and ezetimibe reduced valve replacement risk by 60% in patients with mild stenosis and LDL > 4 mmol/L [50], but in our study none of the patients received such therapy. In the present study, adjusting for cholesterol had no impact on the link between glycaemia and micro-CT parameters, probably due to statin use in the majority of patients.

We also demonstrated that micro-CT parameters were associated with echocardiographic measures of AS severity, further corroborating the findings of our analyses and indicating that micro-CT appropriately reflects valve changes associated with calcification of aortic leaflets. Moreover, micro-CT successfully identified differences in the morphological features of stenotic aortic valves with regard to glucose levels. Since ex vivo micro-CT is known for its high-resolution imaging capabilities and serves as a valuable tool for assessing the structural details of AS calcifications, providing an accurate measurement of tissue calcification [51], this method may be useful in studying the pathomechanisms leading to valve leaflet calcification. In our opinion, improving available in vivo imaging modalities, such as CT with calcium score, to enhance their precision and ability to measure parameters such as CV and TbTh, could provide valuable insights into disease characteristics and progression while facilitating the evaluation of the therapeutic effects of novel agents for the treatment of AS.

### Study limitations

First, the number of individuals in the subgroups was limited, particularly among those with impaired and high glucose levels. However, the study sample represents typical patients with advanced AS in clinical practice and the study was sufficiently powered. Second, all participants were Caucasian, which may limit the generalizability of the findings to other populations. Third, the cross-sectional nature of the study should be mentioned, as HbA_1c_ reflects glycaemic control over the past three months, while aortic stenosis develops over decades. Consequently, the glycaemic status assessed at the time of the study may not fully capture long-term glycaemic control during AS progression, potentially influencing the observed associations. However, some studies suggested that even a single glucose measurement could predict vascular calcification or the risk of AS [28, 33]. Fourth, differences in hemodynamic parameters with regard to glucose levels could be influenced by the time of diagnosis and referral. However, we involved consecutive severe AS patients to minimize selection bias and we found that patients with impaired and high glucose levels consistently exhibited higher PG_max_ and V_max_ compared to individuals with normal glucose.

Moreover, demographic factors, although not statistically significant, could potentially bias the results. However, after adjustment for sex and BMI, the associations between glycaemic status and micro-CT parameters remained significant. Additionally, volume and surface area are heavily influenced by valve size, which could have been estimated using the left ventricular outflow tract diameter; however, this data was not collected as the study was a pilot conducted on a limited number of patients. However, we calculated percentage of calcifications per valvular leaflet sample size. Similarly, body surface area (BSA) was not accounted for in the analysis, and we acknowledge that indexing values to BSA would have improved the interpretability of our findings. Nevertheless, considering the strong correlation between BSA and BMI, a potential error in our calculations is expected to be minimal.

Longitudinal studies in individuals at early stages of AS are essential, with a particular emphasis on monitoring valvular calcification over time using available methods, such as calcium scoring. Finally, since this study focused on participants with severe AS, the results may not be applicable to individuals with mild or moderate forms of the disease.

## Conclusions

Our study showed that the pattern of valvular calcification in AS is associated with glycaemic status and plasma AGEs concentrations. Given that impaired and high fasting glucose is associated with valvular calcification, mineralization, and AS severity, our findings highlight a potential link between pre-diabetes and valve damage, suggesting that strict glycaemic control could be beneficial for this patients group. While our results do not establish a causal relationship, further studies are warranted to explore whether glycaemic control could influence the development and/or progression of aortic valve disease. Moreover, ex vivo micro-CT analysis of valvular calcification in patients with AS provides a precise tool for examining calcification structure in relation to comorbidities, contributing to a better understanding of disease mechanisms.

## Electronic supplementary material

Below is the link to the electronic supplementary material.

Supplementary Material 1

## Data Availability

The datasets used and/or analyzed during the current study are available from the corresponding author on reasonable request.

## Abbreviations

ACE: Angiotensin converting enzyme
AGEs: Advanced glycation end products
AS: Aortic stenosis
AVA: Aortic valve area
BMP: Bone morphogenetic protein
CV: Calcium volume
DM: Diabetes mellitus
HbA_1c_: Glycated haemoglobin
Micro-CT: Micro-computed tomography
LVEF: Left ventricular ejection fraction
NF-κB: Nuclear factor-κB
PG_max_: Maximal transvalvular pressure gradient
PG_mean_: Mean transvalvular pressure gradient
SAVR: Surgical aortic valve replacement
sRAGE: Soluble receptor for AGEs
SV: Surface volume
TAVR: Transcatheter aortic valve replacement
TbTh: Trabecular thickness
V_max_: Peak transvalvular velocity
VICs: Valve interstitial cells
