# Supplementary material for: Dysglycaemia is associated with the pattern of valvular calcification in micro-computed tomography analysis: an observational study in patients with severe aortic stenosis
Source: Cardiovasc Diabetol. 2025 Mar 20;24:129. doi: 10.1186/s12933-025-02691-y (PMC11927127; doi:10.1186/s12933-025-02691-y)
Supplement: Supplementary file 1 — Supplementary Material 1 [file 12933_2025_2691_MOESM1_ESM.docx]

**Supplemental Table 1.** Determinants of micro-CT parameters in patients with aortic stenosis.

| **Factor/variable** | **Univariable**  **β (95% CI)** | **Multivariable***  **β (95% CI)** |
| --- | --- | --- |
| **CV** | | |
| Age, per year | -0.233 (-0.618; 0.149) |  |
| Sex, male/female | -0.254 (-0.609; 0.100) |  |
| BMI, per 1 kg/m^2^ | 0.094 (-0.258; 0.446) |  |
| V_max_, per 1 m/s | -0.027 (-0.419; 0.366) |  |
| Glucose, mmol/l | 0.469 (0.076; 0.863) | 0.505 (0.165; 0.846)* |
| HbA_1c_, per 1% | 0.403 (0.041; 0.764) |  |
| AGEs, ng/ml | 0.361 (-0.007; 0,730) |  |
| **TbTh_mean_** | | |
| Age, per year | -0.074 (-0.383; 0.235) |  |
| Sex, male/female | -0.016 (-0.315; 0.284) |  |
| BMI, per 1 kg/m^2^ | -0.103 (-0.393; 0.187) |  |
| V_max_, per 1 m/s | -0.218 (-0.537; 0.101) |  |
| Glucose, mmol/l | 0.853 (0.517; 1.190) | 0.739 (0.474-1.005)** |
| HbA_1c_, per 1% | 0.714 (0.437-0.990) |  |
| AGEs, ng/ml | 0.543 (0.212-0.875) |  |

Abbreviations: CI, confidence interval; for other abbreviations – see Table 1.

***** adjusted for BMI and sex, adjusted R^2^=0.23

**adjusted for BMI and sex, adjusted R^2^=0.53
